# High prevalence and clonal dissemination of OXA-72-producing *Acinetobacter baumannii* in a Chinese hospital: a cross sectional study

**DOI:** 10.1186/s12879-018-3359-3

**Published:** 2018-09-29

**Authors:** Yong Chen, Yuying Yang, Lin Liu, Guangbin Qiu, Xuelin Han, Shuguang Tian, Jingya Zhao, Fangyan Chen, Hajo Grundmann, Haifeng Li, Jinke Sun, Li Han

**Affiliations:** 1 Department of Hospital Infection Control, Chinese PLA Institute for Disease Control and Prevention, 20# Dongda Str, Beijing, 100071 China; 2 School of Public Health, Shenyang Medical College, Shenyang, China; 3 The 202nd Hospital of PLA, Shenyang, 110003 China; 4 Laboratory of Tropical Biomedicine Technology, School of Tropical Medicine and Laboratory Medicine, Hainan Medical University, Haikou, China; 5 Department of Clinical Microbiology, The 202nd Hospital of PLA, Shenyang, China; 6 Department of Infection Prevention and Hospital Hygiene, Faculty of Medicine, University of Freiburg, Freiburg, Germany; 7 Department of Medical Microbiology, University Medical Center Groningen, Rijksuniversteit Groningen, Groningen, The Netherlands; 8 Department of Hospital Infection Control, The 202nd Hospital of PLA, Shenyang, 110003 China; 9 Chinese PLA 202 Hospital, Shenyang, 110003 China

**Keywords:** *Acinetobacter baumannii*, OXA carbapenemases, Risk factor, Molecular typing, Clone dissemination

## Abstract

**Background:**

Carbapenem resistance in *Acinetobacter baumannii* in China was mainly mediated by OXA-23-like carbapenemases, while OXA-24/40-like carbapenemases were rarely identified. OXA-72 is one variant of OXA-24/40-like carbapenemases. This study aimed to demonstrate the epidemiology and characterizations of OXA-72-producing *A. baumannii* in a Chinese hospital.

**Methods:**

A total of 107 clinical *A. calcoaceticus*-*A. baumannii* (Acb) complex isolates were collected in a Chinese hospital during between 2014 and 2016. These isolates were identified using Vitek 2 system and *gyrB* multiplex PCR. Vitek 2 system was used for antibiotic susceptibility testing. Genes encoding for major classes of carbapenemases were investigated by PCR. Rep-PCR was used for genotyping of all the *A. baumannii* isolates. The risk factors for carriage of OXA-72-producing or OXA-23-producing *A. baumannii* were analyzed through univariate and multivariate logistic regression.

**Results:**

Of the 107 *Acb* isolates collected, 101 isolates (94.4%) and 6 isolates (5.6%) were identified as *A. baumannii* and *A. pittii*, respectively. 78 *A. baumannii* isolates (77.2%) were carbapenem resistant and mainly cultured from intensive care unit (ICU). *bla*_OXA-72_ and *bla*_OXA-23_ genes were identified in 45(57.7%) and 33(42.3%) carbapenem-resistant *A. baumannii* (CRAB), respectively. Multivariate risk factor analyses showed that prior carbapenem usage and nasogastric intubation were significantly associated with carriage of OXA-72-producing *A. baumannii* or OXA-23-producing *A. baumannii*. Rep-PCR analysis showed that 9 and 22 Rep-PCR types were assigned to 78 CRAB isolates and 23 carbapenem-susceptible *A. baumannii* (CSAB) isolates, respectively. A higher diverstiy of Rep-PCR patterns was observed among OXA-72-producing *A. baumannii* isolates than OXA-23-producing *A. baumannii* isolates, but all of them belonged to the same clone complex. MLST analysis suggested that the OXA-72 isolates from this study correspond to CC92/CC2 clone complex.

**Conclusions:**

This study demonstrates high prevalence and potential clonal spread of closely related genotypes of OXA-72-producing *A. baumannii* within a Chinese hospital. Continuous surveillance is necessary to monitor the dissemination of these strains in other healthcare settings to guide infection control policies in order to curb the spread of this bacterium.

## Background

*Acinetobacter* is a gram-negative coccobacillus that has rapidly emerged as one of the most common nosocomial pathogens worldwide [1]. There are currently at least 31 described *Acinetobacter* genomic species [2], of which *A. calcoaceticus*, *A. baumannii*, *A. pittii*, and *A. nosocomialis* are very closely related, and difficult to distinguish from each other by phenotypic properties [3]. *A. calcoaceticus*-*A. baumannii* (Acb) complex has therefore been proposed to refer to these species [2]. Nevertheless, *A. baumannii* is the most clinically relevant and is notorious for its ability to accumulate diverse mechanisms of resistance [4, 5].

The carbapenem class of antibiotics is considered as the last-resort choice when treating *Acinetobacter* infections. However, an increasing prevalence of carbapenem resistance has been observed in clinical *A. baumannii* isolates from many parts of the world. Carbapenem-associated multiclass resistance among 55,330 U.S. *A. baumannii* isolates from The Surveillance Network database has increased from 20.6% in 2002 to 49.2% in 2008 [6]. In China, the resistance rate of clinical *A. baumannii* to carbapenem gradually increased from < 10% in 2000 to > 60% at present [7, 8]. The major mechanism of carbapenem resistance is production of OXA β-lactamases, which are clustered in three major groups, OXA-23-like, OXA-24/40-like, and OXA-58-like [9–11].

OXA-24/40 β-lactamase was first identified in *A. baumannii* from Spain in 1997 [12]. After that, A further 6 enzyme variants have since been discovered, including OXA-72 [11]. Unlike OXA-23-like, OXA-24/40 β-lactamases were less commonly identified in carbapenem resistant *Acinetobacter* spp. isolates [13]. A large surveillance of OXA-type β-lactamase gene clusters for a total of 2880 *Acinetobacter* spp. isolates collected from 23 Chinese provinces found that *bla*_OXA-23-like_ and *bla*_OXA-24/40-like_ genes were identified in 1316 isolates (45.7%) and 11 isolates (0.4%), respectively [14]. Therefore, OXA-24/40-like β-lactamases were only responsible for a small number of carbapenem-resistance isolates in China, their dissemination and epidemiology in healthcare settings deserves further surveillance and investigation.

This study aimed to demonstrate the occurrence, clinical manifestation and genotypic characterizations of OXA-72-producing *A. baumannii* in a Chinese hospital.

## Methods

### Study settings and isolates information

A total of 107 clinical Acb complex isolates from 107 patients were collected at a tertiary-care comprehensive hospital in northeastern China with 1800 beds, from Oct 2014 to Oct 2016. These isolates were recovered from various specimens including sputum, blood, urine, pleural fluid, secretions and throat swab sample. For each patient, only a single colony of the first isolate was selected for subsequent analysis. All the clinical isolates were stored at − 80 °C until use. Data for each isolate and each patient were obtained through review of microbiology lab results and medical records. Risk factor data, including ICU stay, presence of invasive procedures and antibiotic treatment, referred to those which were present before the isolation of the index *Acinetobacter* strains. All data were anonymously collected and interpreted.

### Strain identification and in vitro antibiotic susceptibility testing

Identification of Acb complex isolates was initially performed using automated identification systems the VITEK 2 compact system (BioMérieux, Craponne, France). Further identification of the Acb complex to the species level was performed by *gyrB* multiplex PCR [15, 16].

In vitro susceptibilities to ampicillin-sulbactam, piperacillin-tazobactam, ceftazidime, cefepime, meropenem, imipenem, gentamicin, amikacin, ciprofloxacin, levofloxacin, colistin, minocyline were determined by the VITEK 2 compact system (BioMérieux, Craponne, France). *Escherichia coli* (ATCC 25922) and *Pseudomonas aeruginosa* (ATCC 27853) were used as quality control strains. Results were interpreted according to the Clinical and Laboratory Standards Institute (CLSI, 2016) guidelines.

### Detection of carbapenemases genes

One white loop (1 μl) of 24 h plate culture of *Acinetobacter* bacteria was resuspended in 200 μl of sterilized and DNA free water. The bacterial suspensions were then heated for 10 min at 96 °C and centrifuged 5 min at 13000 rpm. The supernatant was used as the genomic DNA for the following molecular experiments.

Genes encoding for major classes of A, B, and D carbapenemases for all the Acb isolates were investigated by PCR. The presence of *bla*_OXA-23-like_, *bla*_OXA-24-like_, *bla*_OXA-51-like_, and *bla*_OXA-58-like_ genes were detected through multiplex PCR assay [17]. Metallo-β-lactamase encoding genes, *bla*_NDM-like_, *bla*_IMP-like_ and *bla*_VIP-like_, were detected with PCR conditions and primers as previously described [18]. The *bla*_OXA-24-like_ variant was further identified through PCR and DNA sequencing as described [19].

### Molecular typing

Rep-PCR was used for genotyping of all the *A. baumannii* isolates, using the primer pair REP 1(5’-IIIGCGCCGICAGGC-3′) and REP 2(5’-ACGTCTTATCAGGCCTAC-3′) [20]. The conditions for PCR and the gel electrophoresis were the same as previously described [21]. Rep-PCR results of each isolate was compared to all of the other isolates in a pairwise manner, isolates with identical band patterns were considered to be of identical Rep-PCR types. A minimum spanning tree was created to show the differences between patterns through BioNumerics 6.6 software (Applied Maths, Kortrijk, Belgium). The spanning tree was limited to those patterns that differ by a single band, two Rep-PCR types that differ by two or more bands were connected. Nine *bla*_OXA-24-like_ gene positive representative isolates from each different Rep-PCR type were randomly chosen for multilocus sequence typing (MLST) analysis according to ‘Pasteur’ scheme [22].

### A review of literature

Currently, there were two MLST schemes available for *Acinetobacter*. In order to differentiate between the two schemes, STs and CCs were designated as ST^B^/CC^B^ for the Bartual scheme and ST^P^/CC^P^ for the Pasteur scheme. A search of previous published papers giving the MLST results of OXA-24/40-like producing *Acinetobacter* spp. was conducted to illustrate the population structure of OXA-24/40 strains from different countries. The MLST data from ‘Pasteur’ scheme and ‘Bartual’ scheme were separately analyzed by Bionumerics 6.6 and presented as a minimum spanning tree for categorical data with default settings.

### Statistical analysis

Statistical analyses were performed using SPSS 19.0 (IBM, Armonk, NY, USA). The comparisons of patients’ characterics were conducted by chi-square test or Mann-Whitney U test. The risk factors for carriage of *bla*_OXA-72_-positive or *bla*_OXA-23-like_-positive *A. baumannii* were analyzed through univariate and multivariate logistic regression. In multivariate logistic regression, ICU stay and length of ICU stay were not included as they were highly associated with many other predisposing factors, including invasive procedures and antibiotic use. The index of diversity and the 95% confidence intervals (CIs) were calculated as described previously [23]. *P* values < 0.05 are considered statistically significant.

## Results

Of the 107 *Acinetobacter* isolates, 101 isolates (94.4%) and 6 isolates (5.6%) were identified as *A. baumannii* and *A. pittii*, respectively. Five *A. pittii* isolates were susceptible to all the antibiotics tested and one isolate exhibited an intermediate resistance phenotype to minocycline. Among the 101 *A. baumannii* isolates, 78 isolates (77.2%) were resistant to carbapenems (meropenem or imipenem). The rates of resistance to piperacilin/tazobactam, ampicillin/sulbactam, amikacin, gentamicin, ceftazidime, ciprofloxacin and levofloxacin in *A. baumannii* were all above 60%, as most of the carbapenem-resistance isolates exhibited a multidrug resistance phenotype. Four *A. baumannii* isolates were resistant to polymyxin.

The 107 *Acinetobacter* isolates were cultured from 71 male and 46 female patients. Fifty-one isolates were cultured from patients in ICU and all these isolates were carbapenem-resistant. The detection of carbapenemase genes showed that 33 (42.3%) of the 78 carbapenem-resistant *A. baumannii* (CRAB) were positive for the *bla*_OXA-23-like_ gene, while the other 45 isolates (57.7%) were positive for the *bla*_OXA-24/40-like_ gene. All these isolates harboured *bla*_OXA-51-like_ gene. DNA sequencing showed that all the *bla*_OXA-24/40-like_ amplicons belonged to *bla*_OXA-72_ (GenBank accession number MF781069). The *bla*_OXA-58-like_ gene was detected in one carbapenem-susceptible *A. baumannii* (CSAB) isolate, the MICs for imipenem and meropenem were 0.5 μg/ml and 0.25 μg/ml, respectively. None of these *Acinetobacter* isolates were positive for *bla*_IMP-like_, *bla*_VIM-like_ or *bla*_NDM-like_ genes.

Eighty-four (83.2%) of 101 *A. baumannii* isolates were associated with an infection (primarily low respiratory tract infection) and antibiotic treatment, while the other 27 isolates were just colonized. The characterics of 101 patients colonized or infected with *A. baumannii* were shown in Table 1. There were no significant differences over age and gender distribution among patients colonized or infected with CRAB and CSAB strains. More than 60% of the CRAB patients stayed at ICU at the time of bacteria isolation, while only one CSAB patient stayed at ICU.

**Table 1:** Characteristics of 101 patients colonized or infected with *Acinetobacter baumannii* in a Chinese hospital

Characteristics	No. of patients colonized or infected with *A. baumannii*		χ^2^	*P* values
	Carbapenem-resistant (*n* = 78)	Carbapenem-susceptible (*n* = 23)		
Age, median years(range)	77(21,94)	76(2,96)	–	0.463
Male Gender, n(%)	50(64.1)	19(82.6)	2.81	0.094
Length of hospital stay, median days (range)	11(1,3650)	8(1,1400)	–	0.786
ICU stay, n (%)	49(62.8)	1(4.3)	24.30	< 0.001
Associated with an infection, n(%)	67(85.9)	17(73.9)	1.07	0.302
All-cause mortality of patients 14 days after isolation of *A. baumannii*, n(%)	16(20.5)	4(17.4)	0.001	0.974

*ICU* intensive care unit

The results of univariate logistic analysis showed that there were many common risk factors for carriage of OXA-72-producing *A. baumannii* or OXA-23-producing *A. baumannii*, such as ICU stay, mechanical ventilation, nasogastric intubation and carbapenem treatment (Table 2). In multivarite analysis, prior carbapenem usage and nasogastric intubation were significantly associated with carriage of OXA-72-producing *A. baumannii* or OXA-23-producing *A. baumannii.* An additional risk factor, urinary catheter, was also significantly associated with carriage of *bla*_OXA-23-like_-positive *A. baumannii* (Table 3).

**Table 2:** Univariate logistic analysis of risk factors for carriage of OXA-72-producing *Acinetobacter baumannii* or OXA-23-producing *A. baumannii*

Characteristics	Control cases^a^ (*n* = 23)	OXA-72 cases (*n* = 45)	OR(CI 95%)	*P* value	OXA-23 cases (*n* = 33)	OR(CI 95%)	*P* value
Age, median(range)	76(2,96)	78(28,94)	1.02(1.00,1.05)	0.081	78(21,90)	1.00(0.99,1.03)	0.460
Male gender, n(%)	22(75.9)	27(60.0)	0.32(0.09,1.08)	0.067	23(69.7)	0.48(0.13,1.79)	0.484
ICU stay, n (%)	1(4.3)	27(60.0)	33.00(4.08,267.04)	0.001	22(66.7)	44.00(5.23,370.52)	< 0.001
Length of stay in the ICU (days), median(range)	0(0,2)	5(0,100)	2.29(1.20,4.37)	0.012	3(0,100)	2.29(1.03,5.08)	0.042
Predisposing factors							
Urinary catheter	9(39.1)	27(60.0)	2.33(0.84,6.52)	0.106	23(69.7)	3.58(1.17,10.96)	0.026
Mechanical ventilation	2(8.7)	18(40.0)	7.00(1.46,33.59)	0.015	14(42.4)	7.74(1.55,38.56)	0.013
Central venous catheter	5(21.7)	14(31.1)	1.63(0.50,5.26)	0.417	17(51.5)	3.83(1.15,12.74)	0.029
Tracheostomy	1(4.3)	10(22.2)	6.29(0.75,52.56)	0.090	11(33.3)	11.00(1.31,92.63)	0.027
Transfusion	1(4.3)	5(11.1)	2.75(0.30,25.05)	0.369	7(21.2)	5.92(0.68,51.92)	0.108
Nasogastric intubation	4(17.4)	28(62.2)	7.82(2.27,26.91)	0.001	19(57.6)	6.45(1.79,23.19)	0.004
Cephalosporin	12(52.2)	15(33.3)	0.46(0.16,1.27)	0.136	12(36.4)	0.53(0.17,1.55)	0.242
β-lactam/β-lactamase inhibitor combinations	12(52.2)	24(53.3)	2.61(0.90,7.57)	0.077	12(36.4)	1.31(0.42,4.07)	0.645
Quinolone	3(13.0)	19(42.2)	4.87(1.26,18.79)	0.022	10(30.3)	2.90(0.70,12.02)	0.143
Carbapenem	4(17.4)	21(46.7)	4.16(1.22,14.18)	0.023	20(60.6)	7.31(2.02,26.40)	0.002

^a^Control cases: 23 patients colonized or infected with carbapenem-susceptible *Acinetobacter baumannii*

*ICU* intensive care unit

**Table 3:** Multivariate logistic analysis of risk factors for carriage of OXA-72-producing *Acinetobacter baumannii* or OXA-23-producing *A. baumannii*

Characteristics	OXA-72 cases		OXA-23 cases	
	OR(CI 95%)	*P* value	OR(CI 95%)	*P* value
Urinary catheter	–	–	4.94(1.61–21.07)	0.031
Nasogastric intubation	7.65(2.14,27.36)	0.002	7.95(1.79–35.34)	0.006
Carbapenem usage	4.02(1.07,15.06)	0.039	10.05(2.21–45.58)	0.003

**Fig. 1:**
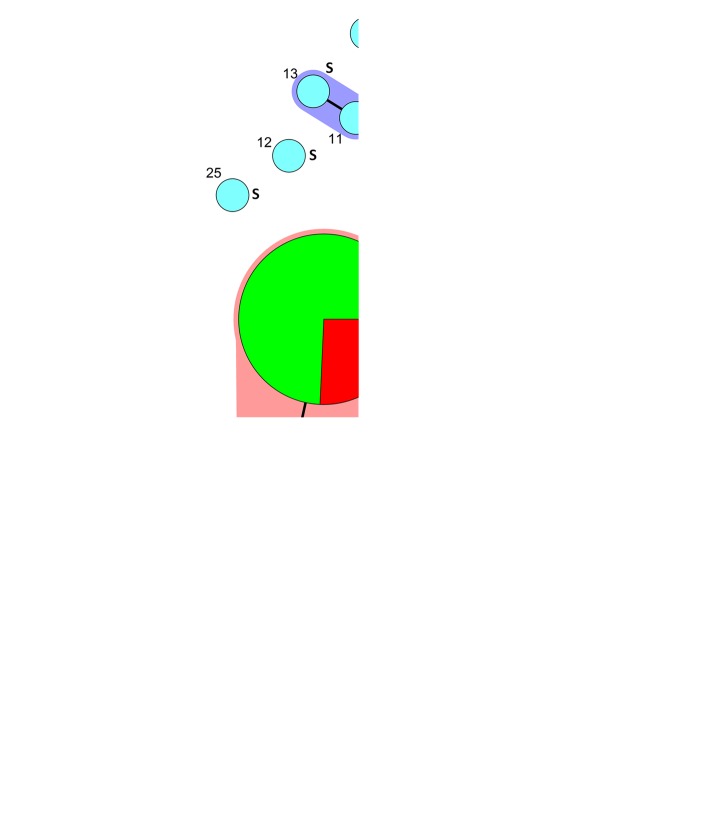
Minimum spanning tree of 101 *Acinetobacter baumannii* isolates based on Rep-PCR patterns. Each circle represents one unique genotype. OXA-72-producing carbapenem-resistant *A. baumannii* (CRAB), OXA-23-producing CRAB and carbapenem-susceptible *A. baumannii* (CSAB) isolates are indicated with red, green, and blue colors, respectively. The size of each circle corresponds to the number of isolates. The lines connecting the circles indicate those patterns that differ by a single band. R: carbapenem-resistant strains. S: carbapenem-susceptible strains

The results of Rep-PCR patterns and corresponding strain information for the 101 *A. baumannii* were shown in Table 4. In total, 9 Rep-PCR types were assigned to 78 CRAB isolates, while 22 Rep-PCR types were assigned to 23 CSAB isolates. The index of diversity (DI) for CRAB was 0.750 (95% CI: 0.671–0.829), which was significantly lower than for CSAB (DI = 0.996, 95% CI: 0.986–1.006). Twenty-six and 7 OXA-23-producing *A. baumannii* isolates were identified as Rep-PCR type 1 and 2, respectively, while 45 OXA-72-producing *A. baumannii* isolates were distributed in the 9 Rep-PCR types. Minimum spanning tree analysis of Rep-PCR patterns showed that all the 78 CRAB isolates were clustered into one clone complex, while most of the CSAB isolates were not connected to each other (Fig. 1). MLST analysis showed that all the 9 representative OXA-72 isolates in this study belong to ST2 (Table 4).

**Table 4:** The Rep-PCR type, MLST type, presence of carbapenemase genes and antimicrobial resistance profile of 101 *Acinetobacter baumannii* isolates

Isolate ID	Rep-PCR type	MLST type	Carbapenemase genes	Antimicrobial resistance profile											
				IMP	MEM	CAZ	FEP	AMK	GEN	CIP	LVX	TZP	SAM	MH	COL
N1312	1	NA	*bla*_OXA-23_	R	R	R	R	R	R	R	R	R	R	S	S
N1314	1	NA	*bla*_OXA-23_	R	R	R	I	R	R	R	R	R	R	S	S
N1316	1	NA	*bla*_OXA-23_	R	R	R	R	R	R	R	R	R	R	S	S
N1318	1	NA	*bla*_OXA-23_	R	R	R	I	S	R	R	R	R	I	S	S
N1352	1	NA	*bla*_OXA-23_	R	R	R	R	R	R	R	I	R	R	S	S
N1359	1	NA	*bla*_OXA-23_	R	R	R	R	R	R	R	I	R	R	S	S
N1362	1	NA	*bla*_OXA-23_	R	R	R	R	R	R	R	R	R	R	S	S
N1369	1	NA	*bla*_OXA-23_	R	R	R	R	R	I	R	I	R	R	S	S
N1371	1	NA	*bla*_OXA-23_	R	R	R	R	R	R	R	I	R	R	S	S
N1383	1	NA	*bla*_OXA-23_	R	R	R	R	R	R	R	R	R	R	S	S
N1384	1	NA	*bla*_OXA-23_	R	R	R	R	R	R	R	R	R	R	S	S
N1386	1	NA	*bla*_OXA-23_	R	R	R	R	R	R	R	I	R	R	S	R
N1395	1	NA	*bla*_OXA-23_	R	R	R	R	R	R	R	R	R	R	S	S
N1396	1	NA	*bla*_OXA-23_	R	R	R	R	R	R	R	R	R	R	S	S
N1398	1	NA	*bla*_OXA-23_	I	R	R	R	R	R	R	R	R	I	S	S
N1401	1	NA	*bla*_OXA-23_	R	R	R	R	R	R	R	R	R	I	S	S
N1407	1	NA	*bla*_OXA-23_	R	R	R	R	R	R	R	R	R	R	S	S
N741	1	NA	*bla*_OXA-23_	R	R	R	R	R	R	R	R	R	R	S	S
N747	1	NA	*bla*_OXA-23_	R	R	R	I	R	R	R	R	R	S	I	S
N748	1	NA	*bla*_OXA-23_	R	R	R	R	R	R	R	R	S	R	I	S
N749	1	NA	*bla*_OXA-23_	R	R	R	R	R	R	R	R	R	R	S	S
N751	1	NA	*bla*_OXA-23_	R	R	R	R	R	R	R	R	R	R	S	S
N752	1	NA	*bla*_OXA-23_	R	R	R	R	R	R	R	R	R	R	I	S
N756	1	NA	*bla*_OXA-23_	R	R	R	I	R	R	R	R	R	S	S	S
N762	1	NA	*bla*_OXA-23_	R	R	R	R	R	R	R	R	R	R	I	S
N764	1	NA	*bla*_OXA-23_	R	R	R	R	R	R	R	R	R	R	I	S
N1304	1	NA	*bla*_OXA-72_	R	R	R	S	R	R	R	I	R	I	S	S
N1307	1	2	*bla*_OXA-72_	R	R	R	I	R	R	R	R	R	I	S	S
N1309	1	NA	*bla*_OXA-72_	R	R	R	R	R	R	R	I	R	R	S	S
N1311	1	NA	*bla*_OXA-72_	R	R	R	I	R	R	R	I	R	I	S	S
N1315	1	NA	*bla*_OXA-72_	R	R	R	R	R	R	R	R	R	R	S	S
N743	1	NA	*bla*_OXA-72_	R	R	R	R	R	R	R	R	R	R	R	S
N745	1	NA	*bla*_OXA-72_	R	R	R	I	R	R	R	R	R	I	I	S
N750	1	NA	*bla*_OXA-72_	R	R	R	I	R	R	R	R	R	R	I	S
N754	1	NA	*bla*_OXA-72_	R	R	R	I	R	R	R	R	R	I	S	S
N1412	2	NA	*bla*_OXA-23_	I	R	R	R	R	R	R	R	R	R	S	S
N1419	2	NA	*bla*_OXA-23_	I	R	R	R	R	R	R	R	R	R	S	S
N1420	2	NA	*bla*_OXA-23_	R	R	R	R	R	R	R	R	R	S	S	S
N1424	2	NA	*bla*_OXA-23_	I	R	R	R	R	R	R	R	R	R	S	S
N1428	2	NA	*bla*_OXA-23_	R	R	R	R	R	R	R	R	R	I	S	S
N1447	2	NA	*bla*_OXA-23_	R	R	R	R	R	R	R	I	R	R	S	S
N1449	2	NA	*bla*_OXA-23_	R	R	R	R	R	R	R	I	R	R	S	S
N1372	2	NA	*bla*_OXA-72_	R	R	R	I	R	R	R	R	R	R	I	S
N1411	2	NA	*bla*_OXA-72_	R	R	R	I	R	R	R	R	R	R	S	S
N1425	2	2	*bla*_OXA-72_	R	R	R	S	R	R	R	R	R	I	S	S
N1446	2	NA	*bla*_OXA-72_	R	R	R	I	R	R	R	R	R	R	S	S
N1451	2	NA	*bla*_OXA-72_	R	R	R	R	R	R	R	R	R	R	I	S
N1348	3	NA	*bla*_OXA-72_	R	R	R	I	R	R	R	R	R	R	S	S
N1349	3	NA	*bla*_OXA-72_	R	R	R	I	R	R	R	R	R	R	S	S
N1368	3	NA	*bla*_OXA-72_	R	R	R	I	R	R	R	R	R	R	S	S
N1376	3	NA	*bla*_OXA-72_	R	R	R	S	R	R	R	R	R	R	S	S
N1378	3	NA	*bla*_OXA-72_	R	R	R	I	R	R	R	R	R	R	S	S
N1421	3	2	*bla*_OXA-72_	R	R	R	I	R	R	R	R	R	I	S	S
N1427	3	NA	*bla*_OXA-72_	R	R	R	I	R	R	R	R	R	R	S	S
N1429	3	NA	*bla*_OXA-72_	R	R	R	I	S	S	R	I	R	R	S	S
N1432	3	NA	*bla*_OXA-72_	R	R	R	I	R	R	R	R	R	S	S	S
N1436	3	NA	*bla*_OXA-72_	R	R	R	R	R	R	R	R	R	R	S	S
N1437	3	NA	*bla*_OXA-72_	R	R	R	I	R	R	R	R	R	R	S	S
N1365	4	NA	*bla*_OXA-72_	R	R	R	I	R	R	R	R	R	R	S	S
N1366	4	NA	*bla*_OXA-72_	R	R	R	R	R	R	R	R	R	R	S	S
N1422	4	2	*bla*_OXA-72_	R	R	R	S	R	R	R	I	R	I	S	S
N1434	4	NA	*bla*_OXA-72_	R	R	R	I	R	R	R	R	R	R	S	S
N1361	5	NA	*bla*_OXA-72_	R	R	R	I	R	R	R	R	R	R	S	S
N1375	5	NA	*bla*_OXA-72_	R	R	R	R	R	I	R	R	R	R	S	S
N1380	5	NA	*bla*_OXA-72_	R	R	R	S	R	R	R	R	R	R	S	S
N1381	5	NA	*bla*_OXA-72_	R	R	R	S	R	R	R	R	R	R	S	S
N1397	5	2	*bla*_OXA-72_	R	R	R	I	R	R	R	R	R	R	S	S
N1400	5	NA	*bla*_OXA-72_	R	R	R	I	R	R	R	R	R	R	S	S
N1350	6	NA	*bla*_OXA-72_	R	R	R	I	R	R	R	R	R	R	S	S
N1353	6	2	*bla*_OXA-72_	R	R	R	R	R	R	R	R	R	R	S	S
N1370	6	NA	*bla*_OXA-72_	R	R	R	I	R	R	R	R	R	R	I	S
N1391	7	2	*bla*_OXA-72_	R	R	R	R	R	R	R	R	R	R	S	S
N1357	8	2	*bla*_OXA-72_	R	R	S	S	S	R	R	R	I	S	S	S
N1374	8	NA	*bla*_OXA-72_	R	R	R	I	R	R	R	R	R	R	S	S
N1379	8	NA	*bla*_OXA-72_	R	R	R	I	R	R	R	R	R	R	S	S
N1392	8	NA	*bla*_OXA-72_	R	R	R	S	R	R	R	R	R	R	S	S
N1409	8	NA	*bla*_OXA-72_	R	R	R	S	R	R	R	I	R	I	S	S
N1382	9	2	*bla*_OXA-72_	R	R	R	S	R	R	R	R	R	R	S	S
N1415	10	NA	None	S	S	S	S	S	S	S	S	S	S	S	S
N1441	11	NA	None	S	S	S	S	S	R	S	S	S	R	S	S
N1443	12	NA	None	S	S	S	S	S	S	S	S	S	S	S	S
N1408	13	NA	None	S	S	S	S	S	S	S	S	S	S	S	S
N1306	14	NA	None	S	S	S	S	S	S	S	S	S	S	S	S
N1414	15	NA	None	S	S	S	S	S	S	S	S	S	S	S	S
N1363	16	NA	None	S	S	S	S	S	S	S	S	S	S	S	S
N1389	17	NA	None	S	S	S	S	S	S	S	S	S	S	S	S
N1405	18	NA	None	S	S	S	S	S	S	S	S	S	S	S	S
N1406	18	NA	None	S	S	S	S	S	S	S	S	S	S	S	R
N1377	19	NA	None	S	S	R	R	R	R	R	R	R	R	S	S
N1390	20	NA	*bla*_OXA-58_	S	S	S	S	S	R	S	S	S	S	S	S
N1448	21	NA	None	S	S	S	S	S	R	R	R	S	S	S	R
N1354	22	NA	None	S	S	S	S	S	S	S	S	S	S	S	S
N746	23	NA	None	S	S	S	S	S	S	S	S	S	I	S	S
N1364	24	NA	None	S	S	S	S	S	S	S	S	S	S	S	S
N1393	25	NA	None	S	S	S	S	S	S	S	S	S	S	S	S
N1351	26	NA	None	S	S	S	S	S	S	S	S	S	S	S	S
N1399	27	NA	None	S	S	S	S	S	S	S	S	S	S	S	S
N1305	28	NA	None	S	S	S	S	S	S	S	S	S	S	S	S
N1387	29	NA	None	S	S	S	S	S	S	S	S	S	S	S	S
N744	30	NA	None	S	S	S	S	S	S	S	S	S	S	S	R
N1423	31	NA	None	S	S	S	S	S	S	S	S	S	S	S	S

*NA* not available, *IMP* imipenem, *MEM* meropenem, *CAZ* ceftazidime, *FEP* cefepime, *AMK* amikacin, *GEN* gentamicin, *CIP* ciprofloxacin, *LVX* levofloxacin, *TZP* piperacillin-tazobactam, *SAM* ampicillin-sulbactam, *MH* minocyline, *COL* colistin, *R* resistant, *I* intermediate, *S* susceptible

**Fig. 2:**
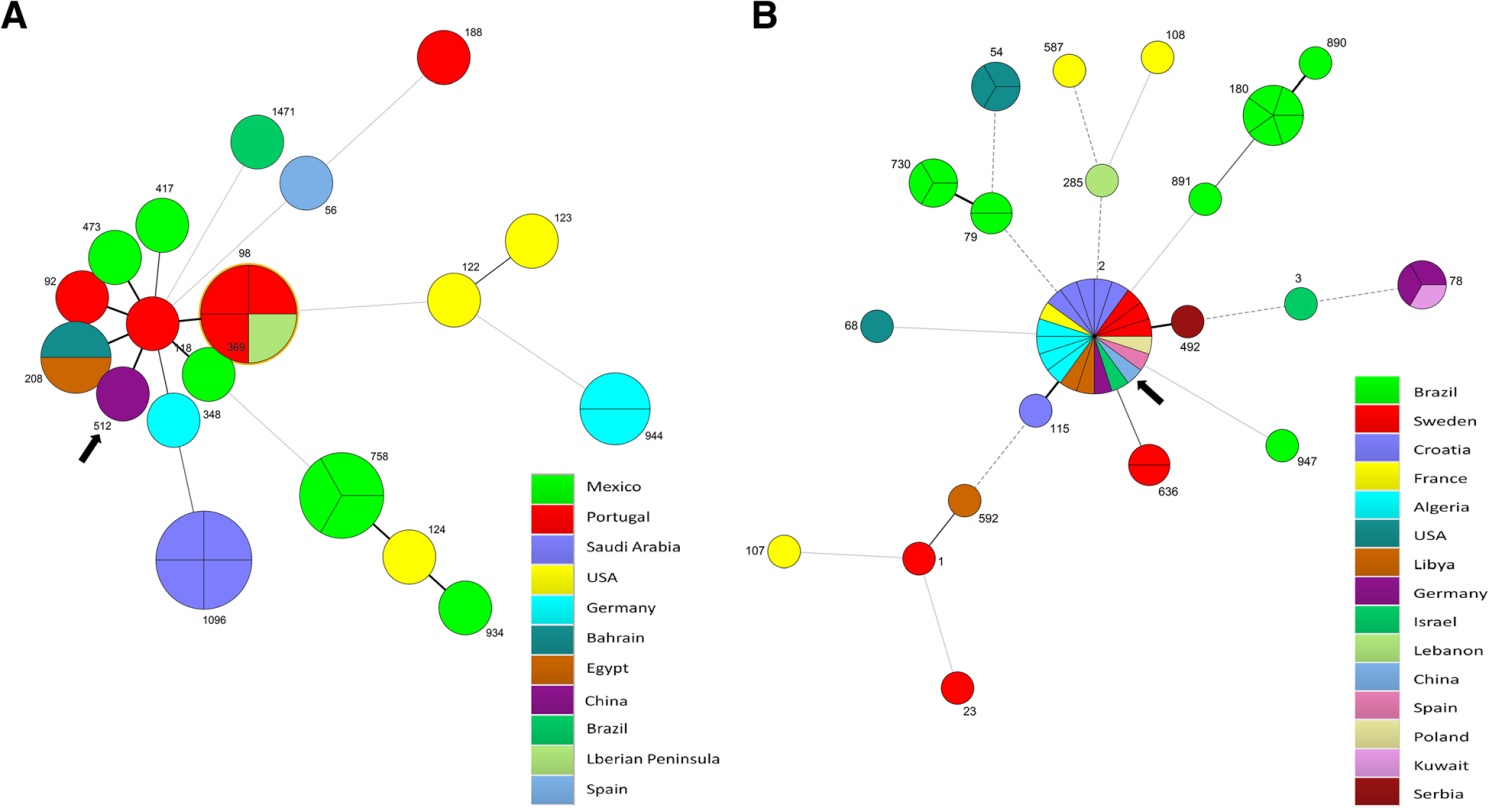
Minimum spanning tree analysis of *bla*_OXA-72_-positive *Acinetobacter baumannii* isolates based on multilocus sequence typing (MLST) data from published literatures. The left panel (**a**) showed the results from ‘Bartual’ scheme, the right panel (**b**) showed the results from ‘Pasteur’ scheme. Each circle represents an independent sequence type (ST). The size of each circle corresponds to the number of isolates. The lines connecting the circles indicate the relationship between different STs. Black arrows are used to indicate the strains originated from China

A literature review of previous published MLST data showed that there were at least 19 ST types (Bartual scheme) for 29 OXA-24/40-like producing *Acinetobacter* spp. isolates from 11 countries and 21 ST types (Pasteur scheme) for 52 OXA-24/40-like producing *Acinetobacter* spp. isolates from 15 countries. Minimum spanning tree analysis of these isolates based on two different MLST schemes was shown in Fig. 2, which suggested that CC92^B^/CC2^P^ represented the predominant clone for the OXA-24/40-like producing *Acinetobacter* spp. isolates from around the world.

## Discussions

Carbapenem-resistant *Acinetobacter* spp. (mainly CRAB) are increasingly recognized as major nosocomial pathogens and considered to be serious threat for human health by US Centers for Disease Control and Prevention and World Health Organization [24, 25]. OXA-23-like carbapenemases were the main reason for the high prevalence and wide dissemination of CRAB from many parts of the world, including China [14, 26, 27]. OXA-24/40-like carbapenemases, which have been reported to be associated with outbreak of nosocomial infection in United States, Spain, Turkey and Ecuador [28–31], accounted for only a small part of CRAB in China [14, 32]. OXA-72, which was first identified in 2004 in an *A. baumannii* isolate from Thailand, belonged to one of the most important variant of OXA-24/40-like carbapenemases [29]. This study reported firstly a high prevalence and clonal dissemination of OXA-72-producing *A. baumannii* in a hospital from northeastern China.

Carbapenem resistance in *A. baumannii* was not significantly associated with 14-day mortality in this study (Table 1), which is in accordance with previous studies [33, 34]. However, carbapenem resistance limits the available therapeutic agents, makes the infection difficult to treat, and might be associated with an additional cost of hospitalization [34]. In this study, there was no significant difference over length of hospital stay for carbapenem resistance and susceptible *A. baumannii*, which might be related with the limited sample size.

In vitro antimicrobial susceptibility testing showed that OXA-72-producing *A. baumannii* and OXA-23-producing *A. baumannii* exhibited similar multidrug resistance profile, suggesting that they could not be differentiated through detection of antimicrobial phenotype. The risk factor analyses implicated that admitted into ICU and length of ICU stay were the most important risk factors for carriage of OXA-72-producing *A. baumannii* and OXA-23-producing *A. baumannii*, as ICU patients are always critical ill and subjected to a lot of risk factors for the acquisition of multidrug resistance organisms (MDROs) [35]. When ICU stay was removed for multivariable analyses, nasogastric intubation and carbapenem use were significantly associated with acquisition of both classes of CRAB, which is in accordance with previous studies [36–38]. The reason for why urinary catheter was significantly associated with carriage of OXA-23-producing *A. baumannii*, but not OXA-72-producing *A. baumannii* deserves further investigation. One possible explanation was that the complex conditions and combined therapy of ICU patients compromised the accuracy of multiple logistics analysis, urinary catheter might be just an indicator for critical ill patients who have a high probability of acquiring certain MDROs through contaminated environment or nursing behavior.

CC92^B^/CC2^P^ was by far the largest and most widely distributed *A. baumannii* clone in the world, especially among OXA-23-producing *A. baumannii* [27, 39, 40]. Although not so widely disseminated, CC92^B^/CC2^P^ was still the most important clone in OXA-24/40-producing *A. baumannii* (Fig. 2). The Rep-PCR and MLST analysis of *A. baumannii* in this study suggested that OXA-72-producing and OXA-23-producing *A. baumannii* isolates were genetically related and belonged to the same clone, CC92^B^/CC2^P^. It seems that OXA-72-producing *A. baumannii* has already become endemic in the ICU since 2014, as most of these isolates were continuously cultured without obvious clustering of isolation time. Enhanced infection control measures, such as hand hygiene education programs, environmental cleaning, antimicrobial stewardship, contact precautions [41], have to be implemented in ICU of this hospital in order to reduce the wide spread of high risk clone, CC92^B^/CC2^P^, which represents the most prevalent clone of CRAB in Chinese hospitals.

There are some limitations for this study. The first is the inclusion criteria of Acb complex strains, it has been demonstrated that a single patient may have more than one genetic type of *Acinetobacter* [42, 43]. To avoid the problem of duplicate data, this study adopted a simple inclusion method of allowing only a single isolate per patient. it might limit the ability to monitor the dynamic changes and complex conditions in patients who may be at particular risk of acquiring antibiotic resistant strains through cross-infection or the development of resistance during antibiotic treatment [44]. Another limitation comes from the design of this study, as this is just an one-center study, the epidemiological characterizations of OXA-72 strains in this study might not be generalized to other healthcare settings in China.

## Conclusions

This study described firstly a high prevalence of OXA-72-producing *A. baumannii* in ICU of a Chinese hospital, which have circulated in this ICU through clonal dissemination for at least two years. Strict infection control measures must be implemented to contain the ongoing dissemination of OXA carbapenemases-producing *A. baumannii* in Chinese ICUs.

## Abbreviations

Acb: *Acinetobacter calcoaceticus*-*A. baumannii*
CC: Clone complex
CRAB: Carbapenem-resistant *A. baumannii*
CSAB: Carbapenem-susceptible *A. baumannii*
DI: Index of diversity
ICU: Intensive care unit
MDROs: Multidrug resistance organisms
MLST: Multilocus sequence typing
ST: Sequence type
